# Brain maps of Iowa gambling task

**DOI:** 10.1186/1471-2202-9-72

**Published:** 2008-07-26

**Authors:** Ching-Hung Lin, Yao-Chu Chiu, Chou-Ming Cheng, Jen-Chuen Hsieh

**Affiliations:** 1 Institute of Neuroscience, School of Life Science, National Yang-Ming University, Taipei, Taiwan; 2 Laboratory of Integrated Brain Research, Department of Medical Research & Education, Taipei Veterans General Hospital, Taipei, Taiwan; 3 Department of Psychology, Soochow University, Taipei, Taiwan; 4 Institute of Brain Science and Brain Research Center, School of Medicine, National Yang-Ming University, Taipei, Taiwan

## Abstract

**Background:**

Somatic Marker Hypothesis (SMH), based on clinical observations, delineates neuronal networks for interpreting consciousness generation and decision-making. The Iowa gambling task (IGT) was designed to verify the SMH. However, more and more behavioral and brain imaging studies had reported incongruent results that pinpointed a need to re-evaluate the central representations of SMH. The current study used event-related fMRI (functional Magnetic Resonance Imaging) to examine neural correlates of anticipation vs. outcome, wins vs. losses, and differential decks' contingencies of IGT.

**Results:**

Behavioral results showed a prominent effect of frequency in driving choices. The insula and basal ganglia were activated during the anticipation phase while the inferior parietal lobule was activated during the outcome phase. The activation of medial prefrontal cortex was especially targeted during the high punishment contingencies. The data suggest that under uncertainty the normal decision makers can become myopic.

**Conclusion:**

The insula and basal ganglia might play a vital role in long-term guidance of decision-making. Inferior parietal lobule might participate in evaluating the consequence and medial prefrontal cortex may service the function of error monitoring.

## Background

Damasio et al. proposed the Somatic Marker Hypothesis (SMH) to interpret the function of the medial prefrontal cortex (MPFC) [1-4]. Earlier neurological studies by Damasio et al. indicated that the MPFC plays an important role in integrating the bodily signals (Somatic Marker), which provide emotional representation of different external events. In another words, normal decision makers with intact MPFC can integrate the bodily signals implicitly and automatically make advantageous real-life decisions, particularly regarding uncertain events which cannot be logically inferred. This is supported by clinical observations that the MPFC patients who retain normal IQ score may still encounter problems in making real-life decisions.

Notably, a growing body of behavioral and theoretical studies of the IGT had pinpointed some possible confounds that may result in misinterpretation of the SMH [5-11]. Some IGT studies suggested that decision-makers may actually preferred bad final-outcome deck B to good final-outcome deck C. Lin and Chiu *et al*. [6,7] termed this phenomenon as "prominent deck B". Wilder *et al*. [9], MacPherson *et al*. [12], Maia and McClelland [8] and Rodríguez-Sa'nchez *et al*. [13] also mentioned that normal decision-makers may be guided by gain-loss frequency rather than final outcome.

According to neurological studies by Iowa group, Bechara and Damasio [14,15] defined additional brain regions for the neuronal network of SMH. Two loops have been postulated: the "Body Loop" and the "As-if Body Loop". The neural substrates of both loops include the MPFC, amygdala, insular cortex (IN), somatosensory cortex (S1), and brainstem nuclei [14]. They suggested that these brain regions constitute the central representation of the somatic signal processing in generating the advantageous decisions. Recently, the Iowa group had enlisted the striatum, anterior cingulate cortex (ACC) and the dorsolateral prefrontal cortex (DLPFC) to extend the neuronal loops of SMH [15].

However, some neuroscientists [5,10,11] pointed out the theoretical flaws of these loops for SMH, e.g., there being little neurological (lesion) evidence of S1 involvement for the SMH [5]. An increasing number of studies demonstrated that deficits of proposed somatic loops do not necessarily affect IGT performance [9,16-21]. Many IGT related studies [9,16-21] provided data incongruent with the proposed neuronal correlates for SMH. Dunn, et al. [22], after thoroughly reviewing the SMH and IGT-related behavioral, physiological, lesion, and brain imaging studies, reported the diversity of the results [23-30] (see also Table 1). The imaging studies revealed that except the MPFC, regions as the anterior cingulate cortex and dorsolateral prefrontal cortex (DLPFC) were highly involved in decision-making processing during IGT, but were not included from the proposed main loops by the Iowa group [14]. The S1 was not activated except in two studies [23,25]. One major limitation is that these IGT brain imaging studies only investigated the central representations for the risk vs. safe or good vs. bad decks. Furthermore, most IGT brain imaging studies focused only on the MPFC or orbitofrontal cortex (OFC) but did not discuss other regions associated with probability learning [31,32] for decision making under uncertainty.

**Table 1:** Summary of functional brain imaging observations in IGT related studies.

	Main loop of SMH	Ernst et al. (2002) (PET)	Ernst et al. (2003b) (PET)	Bolla et al. (2003) (PET)	Adinoff et al. (2003) (PET)	Tucker et al. (2004) (PET)	Bolla et al. (2005) (PET)	Fukui et al. (2005) (fMRI)	Northoff et al. (2006) (fMRI)
**Cortical Areas**									

Medial Prefrontal Cortex (R)	**V**		**V**			**V**		**V**	**V**
Medial Prefrontal Cortex (L)	**V**		**V**	**V**		**V**		**V**	**V**
Orbitofrontal Cortex (R)		**V**		**V**			**V**		
Orbitofrontal Cortex (L)									
Dorsolateral Prefrontal Cortex (R)	**V(Extended)**	**V**	**V**	**V**			**V**		
Dorsolateral Prefrontal Cortex (L)	**V(Extended)**		**V**		**V**				
Middle Frontal Gyrus (R)						**V**			
Middle Frontal Gyrus (L)						**V**			
Superior Frontal Gyrus (R)						**V**			
Superior Frontal Gyrus (L)						**V**			
Somatosensory	**V**								
Inferior Parietal Lobule		**V**							
Insular Cortex	**V**	**V**	**V**						
Anterior Cingulate Cortex	**V(Extended)**	**V**	**V**		**V**	**V**			

**Subcortical Areas**									

Lentiform Nucleus (Basal Ganglia)	**V(Extended)**	**V**							
Amygdala	**V**								
Hippocampus			**V**						
Thalamus		**V**							
Brainstem Nuclei	**V**								
Cerebellum (L)		**V**					**V**		

The table shows divergent results across the brain-imaging studies related to SMH and IGT.

It is noteworthy that the aforementioned studies did not investigate the critical dimensions of "anticipation" and "experience" of the decision making, respectively, to elucidate the complexity of the IGT-brain processing. Knutson *et al*. [33] observed that anticipation of reward and the actual fulfillment of outcome may involve different brain circuitries. During reward anticipation the nuclear accumbens was involved while during outcome experience the MPFC was activated. Moreover, Breiter *et al*. [34], using a gambling task comprising both monetary reward and punishment, demonstrated that anticipation and experience of monetary gains and losses may have different central representations.

In this study, the event-related fMRI (functional Magnetic Resonance Imaging) was exploited to monitor brain activity associated with gain-loss frequency and final outcome, respectively. Brain activity during anticipation (decision driving) and experience (value representation) were deciphered to elucidate the neuronal architectures for the two dimensions in the decision making processing. Detailed analyses of anticipation and experience of gain, loss and draw were conducted. Four choices (2 good and 2 bad final-outcome decks) and the hierarchical changes of value in IGT were detailed to track the reactive brain responses. According to the findings obtained by Wilder *et al*. [8,9,12,13,18,35-40], normal decision makers should prefer decks B and D (high-frequency gains decks) over decks A and C (low-frequency gains decks). The lentiform nucleus (LN, basal ganglia) should be targeted to deal with probability information processing [41]. If the SMH holds, then the MPFC is expected to be activated for the integration of somatic markers for decision making under uncertainty.

## Results

### Behavior data and leaning curve

Behavioral results were similar to those obtained by Wilder *et al*. [6-9,12,13,18,35-40]. Gain-loss frequency rather than final outcome dominated subjects' behavior. Behavioral data confirmed the observations obtained by Lin and Chiu *et al*. [6,7] in which subjects preferred bad final outcome deck B of higher-frequency gain to the other three decks (see Additional file 1). Moreover, the learning curve indicated that the deck B was relatively more attractive than the other three decks throughout the game (see Additional file 2).

### Brain activation during anticipation and experience

**Figure 1:**
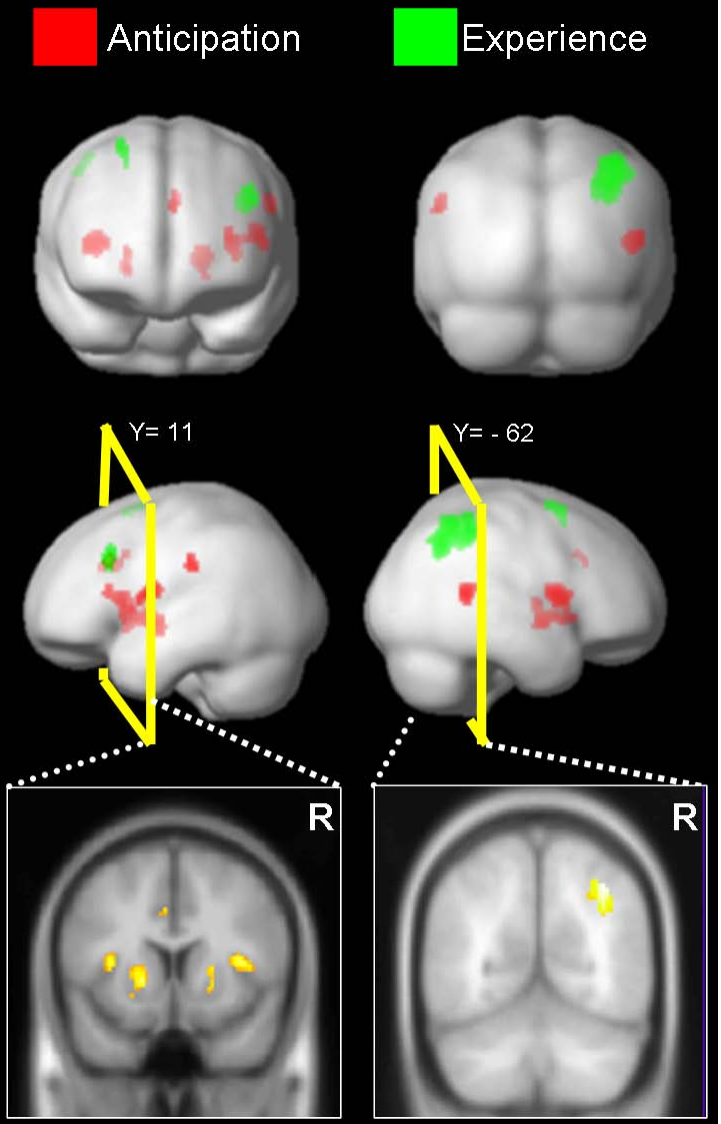
**Brain activation during anticipation and experience**. The red regions (clusters) denoted the brain activation during the anticipation period. The result indicated that the bilateral striatum, insular cortex, superior temporal gyrus and left anterior cingulate cortex were significantly activated (Random Effect: P_*uncorrected *_= 0.0001, K = 30). During the anticipation period (regions mark in red), the insular cortex and basal forebrain rather than the medial prefrontal cortex dominated the generation of subjective feeling and guided the decision-making. On the other hand, during the experience period (regions mark in green), the inferior parietal lobule, superior frontal gyrus and middle frontal gyrus were involved in the consequence assessment of monetary decision (Random Effect: P_*uncorrected *_= 0.0001, K = 30). The thresholds were adjusted for visualization and discussion purpose.

This experiment result demonstrated that IN and LN rather than MPFC was correlated with decision processing, particularly during the anticipation period. This observation supported the hypothesis based on gain-loss frequency. In the original IGT (under the uncertainty game), brain activation during anticipation differed markedly from that during experience (Figure 1). Bilateral IN, LN, right superior temporal gyrus (STG), left inferior parietal lobule (IPL), and ACC were activated during the phase of anticipatory feeling and guide decisions (Table 2). Conversely, the other brain loops related to the experience of monetary outcome encompassed the right IPL [31,42], superior frontal gyrus (SFG) and left middle frontal gyrus (MFG) [43] (Table 3). Brain areas for experience phase varied more than those for anticipation.

**Table 2:** Brain activation during anticipation period.

	**MNI coordinate**					
				
**Brain Region (Hemisphere)**	X	Y	Z	**Cluster Size** **(Voxels)**	**T**	**P _*FWE*-*Corr*_**
Insula (R)	40	6	12	148	7.42	0.008
Lentiform Nuclear (R)	26	-2	-6	119	7.37	0.009
Superior Temporal Gyrus (R)	50	-46	10	76	6.77	0.035
Posterior Insula (L)	-46	-6	14	96	6.65	0.044
Anterior Insula (L)	-34	14	10	67	6.61	0.048
Lentiform Nuclear (L)	-16	12	0	202	6.32	0.080
Inferior Parietal Lobule (L)	-54	-28	30	31	5.90	0.170
Cingulate Gyrus (L)	-4	18	30	32	5.51	0.329

The random effect model was applied with stringent family-wise error correction (thresholded at T = 4.42) and voxel-extent of 30 for presentation brevity and discussion purpose.

**Table 3:** Brain activation during experience phase.

	**MNI coordinate**					
				
**Brain Region (Hemisphere)**	X	Y	Z	**Cluster Size ** **(Voxel)**	**T**	**P _*FWE*-*Corr*_**
Inferior Parietal Lobule (R)	36	-62	44	562	7.68	0.005
Superior Frontal Gyrus (R)	22	10	60	41	5.52	0.294
Medial Frontal Gyrus (L)	-42	22	32	76	5.49	0.309

The random effect model was applied with stringent family-wise error correction (thresholded at T = 4.42) and voxel-extent of 30 for presentation brevity and discussion purpose.

### Brain activation of gain, draw and loss

**Figure 2:**
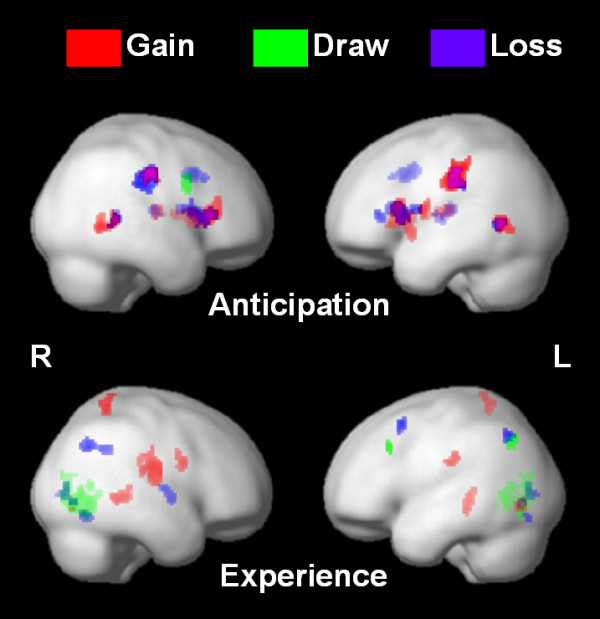
**Brain activation for gain, draw and loss during anticipation and experience**. The red, green and blue represented the gain, draw and loss respectively. The upper figure showed the brain activations in these three conditions during anticipation (Random Effect: all conditions, P_*uncorrected *_= 0.0001, K = 30). Most regions of three conditions overlap during the anticipation stage. The bottom lower figure indicated that different monetary consequences activated different brain regions for different outcomes (Random Effect: Gain: P_*uncorrected *_= 0.0001, K = 30; Draw: P_*uncorrected *_= 0.001, K = 10; Loss: P_*uncorrected *_= 0.001, K = 10. The threshold levels were adjusted for visualization and discussion purpose due to the differences of trial numbers in different conditions, inherent in the IGT.

The respective activation during anticipation and experience of conditions of gain, draw and loss events was analyzed further. The data showed that the brain activations of gain, draw and loss during anticipation and decision processing overlapped considerably, including middle temporal gyrus (MTG), IN, LN (Figure 2).

On the other hand, brain activations of gain, draw and loss following outcome appearance (experience) segregated respectively into different regions, mainly located in the posterior part of the brain. The Precuneus, posterior IN and posterior MTG were engaged in the experience phase. Furthermore, the lateral part of the IPL and left MFG were involved during the outcome phase (experience) [43]. Bilateral MTG were also commonly activated in the three conditions, supporting the fact that the activation of value change is correlated with the bar alteration on the screen (see computer version of IGT [14]).

### Brain activation of decks (A, B, C, D)

**Figure 3:**
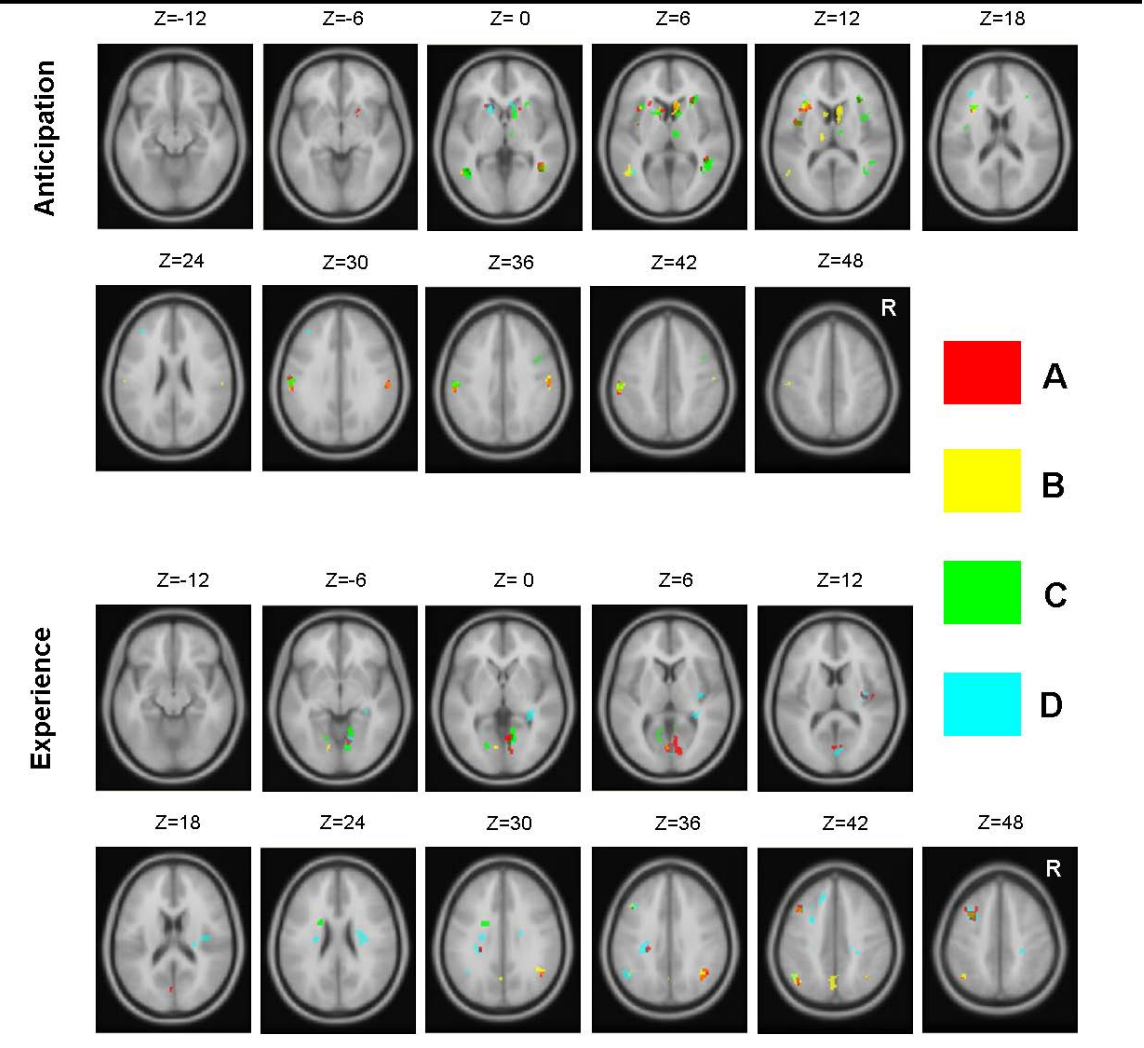
**Brain activation for four decks during anticipation and experience**. The red, yellow, green and blue stand for decks A, B, C, and D, respectively. The upper panel indicated the brain response of the four decks. The activations of four decks mostly overlapped at the bilateral striatum, insular cortex, middle temporal gyrus, thalamus, and primary sensory/motor cortex (Random Effect: all conditions, P_*uncorrected *_= 0.0001, K = 30). The left S1/M1 is more active before choice than the right S1/M1, possibly related to the right hand movement and joystick control. Following card-selection, bilateral inferior parietal lobule, insular cortex, caudate nucleus, posterior cingulate cortex, frontal eye field and primary visual cortex (V1), as well as right superior frontal gyrus were activated (Random Effect: all conditions, P_*uncorrected *_= 0.0001, K = 30). These representations may imply that the attention system is strongly targeted to evaluate the consequences of decks and logically reasoning.

During the anticipatory phase, activation was mostly found in MTG, caudate nucleus (CN), IN, and thalamus across, although not in all, the four desks. The respective brain activation map for each deck overlapped substantially during the anticipatory phase. IN engagement was the only structure identified to be consistent with the originally proposed main loops of SMH (Figure 3).

During the experience phase, diverse patterns for the four decks were revealed. The MFG and IPL showed common activation to the outcomes of all decks. The left Precuneus and the right IPL were activated only during the experience phases of deck A and B (a relatively large gain-loss and bad long-term outcome). The CN tail was activated by the deck A, C, and D, but not by B (Figure 3). The left Precuneus was engaged only by deck B.

### Time courses of regional activity for 11 monetary values ($ +100, $ + 50, ⋯, $ – 350, $ -1150)

**Figure 4:**
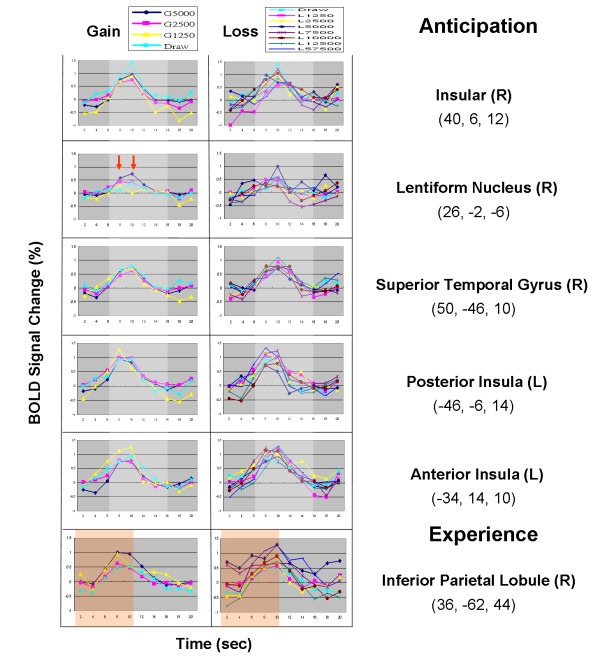
**BOLD response for each monetary value in anticipation and experience**. This figure details the BOLD signal changes (peri-stimulus time-activity curve) in response to each monetary value (original value * 50 for New Taiwan dollar currency) at the activated voxels with local maxima identified during the periods of anticipation and experience of all trials (see Tables 2 & 3, Figure 1). The white shadow indicates the period for anticipation modeling; the pink shadow indicates that of experience modeling. Only the left lentiform nucleus demonstrates the hierarchical signal changes corresponding to the monetary value of the gain at the two seconds after card-turning.

**Figure 5:**
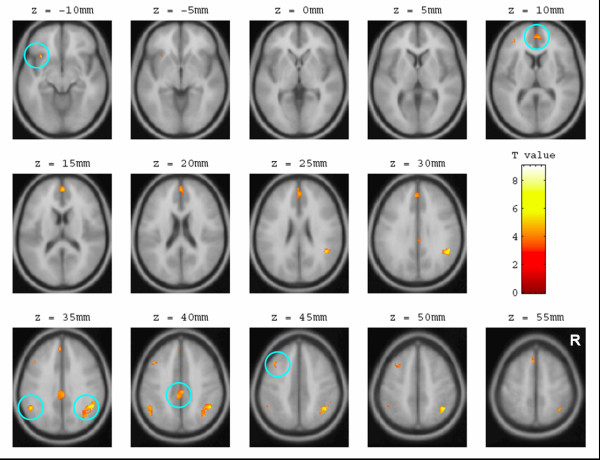
**BOLD response of the medial prefrontal cortex under large loss ($ -1150) condition of deck B**. The detailed analysis for each monetary value found the significant activation (Random Effect: P_*uncorrected *_= 0.001, K = 30) of medial prefrontal cortex during the experience period of large loss condition. Bilateral inferior parietal lobule and posterior cingulate cortex, left frontal eye field, supplementary motor area, and insular cortex were also activated.

The time courses of brain activity for each monetary value as extracted from significantly activated regions (identified from the anticipation- and experience-related maps; 5 from the former and 1 from the latter) were depicted using a peri-stimulus time histogram (PSTH; a default function of SPM2) (Table 2 &3; Figure 4). The activities of most involved regions didn't follow the monetary amount, except for differential responses observed in the right LN and left STG during the anticipation period (Figure 4). MPFC activation was only discovered during the outcome period of $ -1150, which is the largest loss in IGT (Figure 5).

## Discussion

### The "prominent deck B phenomenon"

The perseverated behavior of "prominent deck B phenomenon" was confirmed as previously reported [6-9,12,13] albeit that the effect was sub-significant in our current study (see Additional file 1 and 2). This result corroborated many IGT-related studies that reported that subjects actually preferred bad final-outcome deck B of high-frequency gain over good final-outcome decks C or D [6,8,9,13,18,22,35-40,44,45]. Even Sevy et al., Johnson et al. and Bechara [46,47] also demonstrated the same "prominent deck B phenomenon" which was at odds with their first report [48]. This phenomenon astonishingly contradicts the basic assumption of IGT which posits that normal decision makers are prone to avoid the bad deck. In fact, increasing number of studies consistently showed that participants preferred decks B and D to decks A and C [9,13,18,35-40,44,45]. The reason that the earlier studies failed to demonstrate such phenomenon is that most studies summed the chosen numbers of deck A and B or C and D ad hoc, respectively, for inference on the bad vs. good decks. Notwithstanding, most IGT brain-imaging studies did not report in details the subjects' behavioral data in regards to the preference to four-decks neither.

A few studies out of over one hundred using the similar four-deck format and addressing the chosen frequency had consistently demonstrated that the high-frequency gain decks B and D were preferentially selected than decks A and C in the control group [6,8,9,13,18,22,35-40,44,45]. The "prominent deck B phenomenon" indicated that subjects were overwhelmed by the high-frequency gain of decks B, D, and also C (as opposed to A, C has many standoffs in net value within a trial, see also Chiu et al. [7]) and prohibited by the high-frequency loss of deck A. Nevertheless, it still stands that subjects prefer the good final outcome decks (summation of decks C and D) to the bad final outcome decks (summation of deck A and B) albeit the fact that the subjects may actually be implicitly guided by gain-loss frequency instead of long-term outcome [6]. Such interpretation may contradict the SMH reasoning, however, the gain-loss preponderated choice behavior is not uncommon in the literature on decision making, suggesting that decision makers are often myopic to long-term outcomes [49-51], even under situations of relative certainty [52].

### Brain activation for anticipation and experience

In the current study, the IN and LN were strongly associated with events of uncertain situation. The observation does not completely support the basic assumption of the IGT or SMH neural loops [14] which did not posits that LN participates in the anticipation of uncertainty (Figure 1). The conjoined activation of these two structures might reflect that positive and negative emotions were simultaneously invoked [53] by this uncertain monetary game, which led to a higher arousal state [54-56]. The arousal might catalyze the subject into a self-sustained situation where better psychosomatic condition can be tuned to augment the explorative intent when confronted with a challenge of uncertainty, which in turn can be indispensable for the survival of an organism. Numerous studies have demonstrated that activation of LN is correlated with the "expectation" of reward [33,56-62] and habit learning [41,63] while IN have been reported to be engaged in most studies on visceral and aversive disposition [64-69]. LN can be critical in attributing the positive emotion of anticipation [70,71] and motivation, i.e., driving force [34,61]. On the other hand, IN is not only frequently associated with generation of aversive sensation, e.g., disgust as induced by the repugnant stimuli [67,68], but also related to the fear [72] and addition behavior preservation [73]. The engagement of LN, a neural substrate important for probability learning [41,74] echoes the behavioral results as compelled by the gain-loss frequency.

Following the appearance of outcomes, the activity of LN and IN (limbic structures), subsided and the overall activation pattern eminently changed. The circuitries involve only neocortical areas, mainly encompassing the frontal-parietal network (IPC, MFG, and SFG) for processing value representation and logic inspection. The differential segregation of brain representation in respect to driving force and experience sentiments during anticipation and experience [61] (Figure 1) might attest to two systems, e.g., wanting and liking systems [55,75] to further the elucidation of neural correlates of decision making and the accompanied interoceptive feeling.

### Brain activation for gain, loss and draw conditions

Echoing the behavioral result where the subjects' choices were implicitly biased by high-frequency gain and not hunched by the long-term outcome, the almost common activation pattern in general (Figure 2) during the anticipation phase could not decipher or predict the eventual outcome of gain, loss, or draw conditions. LN, IN, thalamus and MTG were commonly involved in the anticipation phase of three conditions [61]. However, the overlapped activation patterns diverged into differential expressions during experience period. The patterns collectively engaged primary visual cortex (V1), bilateral IPC, SFG, posterior IN and CN [61] where different combinations of neural substrates were noted for respective condition. The result implies that the organism may entail subcortical/limbic system rather than logic system (neo-cortex) to deal with the uncertain situation. On the other hand, after the gain, loss and draws were clearly presented to subjects, the logic system (neo-cortex) was recruited to process these consequences. Our results were inconsistent with cardinal propositions of SMH that posits the MPFC crucial in generating the "guts" feeling and biasing the decision during anticipation (the "hunch" processing in Damasio's term).

### Brain activation for decks

Anticipation commonly activated bilateral CN and anterior IN despite some subtle differences in the activated foci. This was consistent with the previous findings according to gain, loss and draw condition (see Figure 2) preconceived with reward-punishment expectation. The basal forebrain activation (CN in our study) was engaged commonly across the four decks and suggested a cognitive component of probability processing or habit establishment during the task performance. MTG activation may be related to eye scanning among the four decks while the activation of postcentral gyrus (S1) and precentral gyrus (M1) could be attributed to joystick operation. Our findings disagreed with the prediction of SMH that the MPFC, amygdala, IN and S1 subserve primarily the processing of somatic signal for decision.

V1, IPL and SFG were commonly activated during the experience period in addition to other discrete regions under different deck categories. The neo-cortices (frontal-parietal circuitries) were targeted for information processing related to outcome. The IGT is extremely complex and is designed to prevent a subject using the logic reasoning to find out the long-term benefit. The IGT suggests that uncertainty can foster the somatic marker (emotion) system to harbor decision makers in reaching the foresighted (rational) status based on long-term processing. This view is contentious and may be inconsistent with the numerous studies of "emotion", a phenomenon considered irrational, uncontrollable, and immediate [76,77]. The transition from more limbic (anticipation period) to more cortical (experience period) structures did not warrant a better rational behavioral performance in our current study. Furthermore, the behavioral results and the brain activation maps of current study did not demonstrated substantial differences within and between the good decks (C, D) and bad decks (A, B). The "instability" or inconsistent data of IGT across different studies at behavioral or physiological levels [16-21,78] may pinpoint a possible need for a reconsideration of neuro-physiological and neuropsychological assumptions of SMH.

### The role of medial prefrontal cortex in IGT

Damasio [4] stated that "*... the brains of the normal subjects were gradually learning to predict a bad outcome, and were signaling the relative badness of the particular deck before the actual card-turning*." (Damasio, 1994, p. 220). Damasio et al. [1-3,48] suggested that the medial prefrontal cortex (MPFC) plays an important role in integrating bodily signals (Somatic Marker), which provide emotional representation of different external events. Normal decision makers with intact MPFC should be able to integrate the bodily signals implicitly and automatically make advantageous real-life decisions. This is of particular importance under the circumstance in face of uncertain events that cannot be logically inferred. Based on the findings of galvanic skin conductance studies of SMH, it is conceivable that the medial prefrontal cortex (MPFC) should be more activated before selecting the bad decks A or B, namely the anticipation period.

However, the current study did not observe any significant activation under these conditions. Only in the experience period of the largest loss ($ -1150) of desk B that the MPFC was expressed (Figure 5). The MPFC function may contribute to the immediate and shortsighted view as related to error detection [79-87]. The results of this study imply that MPFC may play a critical role in error monitoring or conflict detection [79,88-91], but might not solicitly play a role in the integration of somatic markers (bodily responses) and guiding decisions rationally in the long-run. Further studies are needed to exam this speculation since the results cannot discern the different statistical power calculated from different numbers of trials for each monetary value. For example, the "$ 100 and $ 50" comprised over 70% of trials, but "$ -1150" comprised only 1–3% for each (subject) run. This problem is inherent with the IGT design.

Notably, the right IN manifested the greatest BOLD signal response during the standoff condition. We speculated that the IN may be related to the calibration of mental account or emotion state. On the other hand, right LN corresponded to the hierarchical change of gain, and not to loss in IGT (Figure 4, more activity in NT$ 5000, NT$ 2500 and see Additional file 3) in face of uncertainty. This observation similar to many studies on risk of relative certainty [32-34,57-62,92,93] and is congruent with the view that basal ganglion is related to biological reward system.

## General discussion

This study aimed to disclose neural correlates involved in decision-making processing during anticipation and experience period of the IGT. There are several points that mandate more comprehensive studies for clarification in the future. For example, testing of final-outcome, gain-loss frequency, and prominent deck B phenomenon at the behavioral level reveal only sub-significance. The limited findings of behavioral data may result from the internal variation of IGT and emotions interfering with the performance of the MR-scanning procedure. Whether there exits gender difference of brain activation patterns is of pertinent interest. For instance, Bolla *et al*. reported a gender difference in the activity of OFC and DLPFC during IGT using a gender-balanced approach [94]. Nevertheless, regions such as the basal ganglia [95] and parietal cortex [31,32], suggested to be involved in reward-based probability learning [41,74] under uncertainty, was not emphasized by Bolla et al. study. On the other hand, the negative finding of the ventro-orbital MPFC in this study could possibly be attributed to the susceptibility signal loss of fMRI acquisition.

Our behavioral and imaging findings differed from previous IGT-fMRI studies. Caroselli *et al*. [35] investigated a large population (seventy-one male, sixty-three female) demonstrated also the "prominent deck B phenomenon" and absence of learning curve. A careful review of IGT-fMRI studies disclosed that only Northoff *et al*. [29] presented detailed behavioral data for a four-deck format and demonstrated the "prominent deck B phenomenon". Albeit the discrepancy in the behavioral data with original IGT, the MPFC was found to be activated. The IGT-fMRI study by Fukui *et al*. presented behavioral data for the IGT with a two-deck format [25] and perfectly replicated the original findings as reported by the Iowa group's study in 1994 [48]. However, Fukui *et al*. behavioral data contradicted with a recent study by Sevy *et al*., and Johnson et al., and Bechara on the "prominent deck B phenomenon" [46,47]. Tanabe *et al*. utilized a modified IGT, not original IGT [96], and focused on the functional discrepancies of the ventral MPFC between substance users and a control group. These studies mainly focused on brain activity changes of the MPFC and OFC in either good-bad or risky-safe conditions (two deck format) without discussion on the basal ganglia and parietal cortex in decision-making processing. Notwithstanding, no information was tailored to the "prominent deck B phenomenon". Recently, Chiu et al. [97] used a modified IGT, namely the Soochow Gambling Task (SGT) which possess a relative simple and balanced gain-loss structure than IGT, but keeps all uncertain characteristics of IGT. The experiment result of SGT clearly demonstrated that decision makers' choice was dominated by the gain-loss frequency rather than final-outcome. The heterogeneity of the experimental paradigms and discrepancies of the resulting behavioral data as well as the different approaches for fMRI data analysis command a need to further profound and comprehensive studies for an empirical account for the SMH theory and MPFC function. All the existing behavioral and brain imaging literature actually pinpointed that IGT can be not that optimal to validate the SMH. Therefore, the VMPFC engagements by the IGT remain elusive and mandate a further clarification.

## Conclusion

Our data indicated that both LN [41,56] and IN [68,98] can be targeted for decision-making during anticipation of decision-making under uncertainty. IPL and SFG could be involved in the appreciation of the consequences of choices. Common brain processing might occur during the anticipation periods of gain, loss and draw conditions, but the varied outcomes may predispose different neural substrates for the various experiential dimensions. A similar transition also occurred in the four-deck conditions. The MPFC was founded to be activated only during the experience period of the largest loss of deck B, but not during anticipation period. The original proposition of SMH that MPFC should be implicitly engaged under ambivalence to monitor and inhibit the selection of the bad final-outcome deck by the decision makers is not supported by the current study. This study suggests that under the confrontation of uncertainty the normal decision makers can become myopic. LN and IN might play more vital role in generating the "feeling" accompanying anticipation which in turn can drive for final decision while MPFC may serves more the function of online monitoring and error detection. Consequently, the involvement of consciousness in guiding the decision will be very important in understanding the mechanism of covert (emotion) – overt (rational) coupling [99].

## Methods

### Subjects

Twenty-four college and graduate school students, 19 to 32 years old (Mean age = 21.0 years old; SD = 3.1; 8 males, 16 females) participated in this study. They had not played the IGT before. Written informed consent was obtained from each subject prior to the experiment. Prior to the experiment, each subject gave informed consent to the experimental protocol, which had been approved by the Institutional Ethics Committee of Taipei Veterans General Hospital. The experiment was conducted in accordance with the Declaration of Helsinki.

### Paradigms

**Figure 6:**
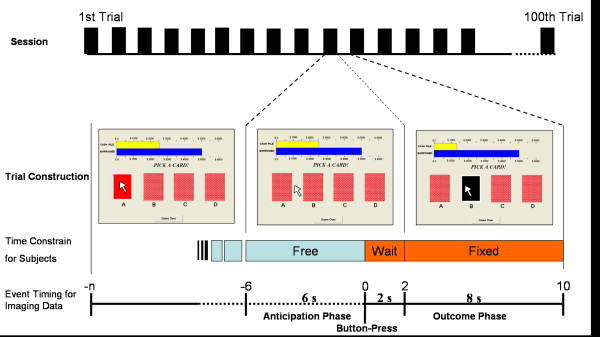
**Computer version of IGT and event-related fMRI design**. Participants were free to consider which deck they wished to choose. The anticipation period encompassed 6 seconds prior to the button press (0 second) for fMRI data. The experience period started from button press. The timing of each selection was jittered by subject's free contemplation. Each subject's time-specific selection pattern was used to model the fMRI signal. After the button-press, the computer displayed the monetary value of gain, loss, or draw via changes in the top bar. The computer displayed the consequences of choices for 8 seconds, during which the participants were unable to press the button.

Each subject was asked to play a computerized four-deck IGT game according to the original design [48]. Subjects were asked to turn a card from the 4-decks voluntarily. The inside of the card was either in black or red, which was unrelated to gain or loss. The subjects were instructed to maximize gains and minimize losses when playing the game. The IGT contained four decks. Decks A and B had potentially large immediate gains ($ 100) and losses ($ -50 to $ -1150) in each trial and a disadvantageous final-outcome ($ -250) from the average of ten trials. Decks C and D had small immediate gain ($ 50) and loss ($ -25 to $ -200) and an advantageous final-outcome ($ 250) (for detailed gain-loss structure and instructions for IGT, please refer to the references [48,100,101]). Subjects did not know the internal IGT structure of gain and loss. The game ended with a total of 100 trials. Different card-display arrangement (ABCD, BCDA ...etc.) for each subject was implemented to counterbalance the card position effect and possible confound of eye movement. Subjects were instructed to play the game using real monetary value (New Taiwan dollar, NT) and were rewarded with their final winnings (divided by 1000). For example, a subject with an account balance of NT 320,000 at the end of the game was rewarded with 320 NT dollars (~US$ 10). The time interval of 6 seconds preceding the button press was defined as anticipation period and the interval after was defined as experience period (see Figure 6 and legend for details).

Subjects conducted a short practice version of IGT for familiarization (with different internal structure of gain and loss) before actual scanning and during the tri-pilot scanning inside the MRI-scanner. The fMRI inter-trial interval was set to be longer than 10 seconds to increase the sensitivity of event-related design [101]. Subjects were free to select the card without time constraint. To avoid the MRI-signal inhomogeneity and the inter-session confounds, the experiment was devoid of 9 interruptions as the original IGT game where the subjects were asked two questions after each 10 trials [8,102-104]. Subjects used a joystick with a press-button for card selection. All subjects completed the fMRI-experiment in less than 1 hour. The structural and anatomical images were acquired after a 15 minute break outside the scanner.

### fMRI data acquisition

Images were acquired using a 3.0 T Bruker MedSpec S300 system MRI (Kalsrube, Germany) with a quadrature head coil. The subjects' heads were immobilized with a vacuum-beam pad in the scanner. Functional data were acquired with a T2*-weighted gradient-echo EPI using BOLD contrast (TR/TE/θ = 2000 ms/50 ms/90°, slice thickness = 5 mm, interslice interval = 1 mm, Field of view (FOV) = 230 × 230 mm^2^, 64 × 64 × 20 matrix, whole brain coverage). The first five images (dummy images) of each session were discarded from the analysis to eliminate possible non-equilibrium effects of magnetization. The anatomical image was acquired using a high-resolution T1-weighted, 3D gradient-echo pulse sequence modified driven equilibrium Fourier transform (MDEFT); TR/TE/TI = 88.1 ms/4.12 ms/650 ms, slice thickness = 1.5 mm, 256 × 256 × 128 matrix, FOV = 230 × 230 mm^2^.

### Data processing and analysis

The computer version of IGT (programmed with Matlab 6.5) was used to register the behavioral profiles and decision time-points. Image and statistical analyses were processed with SPM2 (Welcome Department of Cognitive Neurology, London, UK) and displayed using xjView 4.0 (Human Neuroimaging Lab, Baylor College of Medicine, Houston, TX, U.S.A). Scans were realigned, coregistered, normalized, time corrected and spatially smoothed with a 4 mm full-width-at-half-maximum (FWHM) Gaussian kernel using standard SPM methods. Because of the free choice and event related design, each subject performed 100 events (choices) in total and the scan (image) number ranged from 547 to 652 (scans). General Linear Model (GLM) was first used to model the event-time course for each subject. Regional differences were statistically thresholded at corrected (family-wise error correction) P_*FWE*-*Corr *_= 0.05, voxel extent (K) = 30 in within-subject analysis. A random-effect model was applied for the final second-level (between-subject) analysis (P_*uncorrected *_= 0.0001, K = 30). Coordinates of Z(T)-maxima were registered in Talairach and Tournoux's 3-D brain stereotaxic system under MNI template in the SPM [105]. The images were displayed in neurological convention (the left hemisphere in the image is the left hemisphere of subject).

We shifted forward the hemodynamic response function (hrf) by 6 seconds (3 scans) as indexed to the button press and the epoch ("hrf (with time derivative)" in SPM) was modeled as pre-choice event for anticipatory phase. The epoch backward ("hrf (with time derivative)" in SPM) as indexed to the button press was modeled as post-choice event for experience phase. The experimental designs for the four types of comparisons were listed below: 1) total events: *anticipation- and outcome-related brain activation across all subjects*; 2) gain-loss status: *gain, draw, and loss-related brain activation across all subjects*; 3) deck category: *A, B, C, and D-deck related brain activation across all subjects*; 4) time course of brain activity according to monetary value in NT$ (US$): *5000(100), 2500(50), 1250(25), 0, -1250(-25), -2500(-50), -5000(-100), -7500(-150), -10000(-200), -12500(-250), -57500(-1150)*. Cerebral activity corresponding to each monetary value was extracted from the mostly-activated voxel out of regions identified in brain activation maps from 1). Activity readouts from trials of the same monetary value (not same amount of trials across all monetary values) in each individual were first averaged respectively and then averaged across 24 individuals (a few conditions were averaged with a relatively small number of subjects). Brain activity maps for respective anticipatory and experience phases of each monetary value across 24 subjects were also obtained. For brevity and discussion purpose, we particularly focused and presented the findings on the MPFC, i.e., medial prefrontal cortex.

According to the original design of IGT, the IGT is highly uncertain in comparison to other gambles. Each subject's choice pattern is very different from those of other subjects because subjects possess variant choice numbers and schedules for specific event (e.g., -1150). The time point of a specific event was retrieved from behavioral data to register the brain signal of a specific event. xjView 4.0 was exploited for the result display.

## Abbreviations

ACC: Anterior Cingulate Cortex; CN: Caudate Nucleus; DLPFC: Dorsolateral Prefrontal Cortex; IGT: Iowa Gambling Task; IN: Insular Cortex; IPL: Inferior Parietal Lobule; LN: Lentiform Nucleus; MPFC: Medial Prefrontal Cortex; MFG: Middle Frontal Gyrus; MTG: Middle Temporal Gyrus; OFC: Orbitofrontal Cortex; SGT: Soochow Gambling Task; SMH: Somatic Marker Hypothesis; STG: Superior Temporal Gyrus.

## Competing interests

The authors declare that they have no competing interests.

## Authors' contributions

CH and YC had contributed equally to the idea innovation, literature review, data interpretation and drafting preliminary manuscript. CH and CM had conducted all the data acquisition, and CM was responsible for the MRI scanning. CH worked independently on analyzing behavioral and image data as well as manuscript drafting. CH was co-tutored by YC (behavioral decision and theoretical mainframes) and JC (brain imaging and neurophysiology) for his PhD thesis. JC established all the imaging-experiment setup and finalized the fMRI interpretation and manuscript with CH. All authors gave final consent for manuscript submission and publication.

## Supplementary Material

Additional file 1 Most subjects seem prefer to choose deck B which is consistent with the observation in some researches which demonstrated their IGT data in a clear "four-deck format". Here we provided a repeated measurement ANOVA for two variables (expected value: bad (A, B) vs. good (C, D); gain-loss frequency: high-frequency gain (B, D) vs. Low-frequency gains (A, C)) were listed as below table. The result indicated there is non-significant effect in the testing. Click here for file

Additional file 2 The learning curve of five blocks in each 20 trials showed that subjects gradually chose the deck B and avoid deck A, but there have no obviously ascending pattern for decks C and D in the IGT. The present result may be inconsistent with the original finding of IGT, but might be congruent with most IGT related studies which did not show the learning curve of each deck (most of them use the combination of good decks or bad decks). The repeated measurement ANOVA for three variables (expected value: bad (A, B) vs. good (C, D), gain-loss frequency: high-frequency gain (B, D) vs. Low-frequency gains (A, C), and block (1–5)) was provided here. The result indicated there is a significant effect on block testing. Click here for file

Additional file 3 **Mean response of brain area with value**. The mean activation was further processed from Figure 5, which is the average BOLD signal from the PSTH. The red brackets marked the possible brain regions activated following the monetary change. The lentiform(R) may be in response to the change of positive value. In contrast, the superior temporal gyrus may be sensitive to the change of negative value. Click here for file
