# Supplementary material for: Brain maps of Iowa gambling task
Source: BMC Neurosci. 2008 Jul 26;9:72. doi: 10.1186/1471-2202-9-72 (PMC2518922; doi:10.1186/1471-2202-9-72)
Supplement: Additional file 1 — Most subjects seem prefer to choose deck B which is consistent with the observation in some researches which demonstrated their IGT data in a clear "four-deck format". Here we provided a repeated measurement ANOVA for two variables (expected value: bad (A, B) vs. good (C, D); gain-loss frequency: high-frequency gain (B, D) vs. Low-frequency gains (A, C)) were listed as below table. The result indicated there is non-significant effect in the testing. [file 1471-2202-9-72-S1.doc]

**Additional file 1**


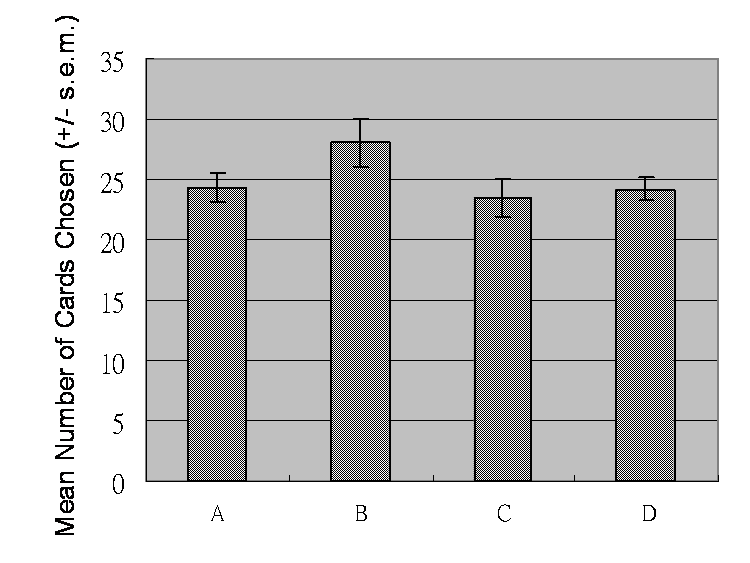


Most subjects seem prefer to choose deck B which is consistent with the observation in some researches which demonstrated their IGT data in a clear “four-deck format”. Here we provided a repeated measurement ANOVA for two variables (expected value: bad (A, B) vs. good (C, D); gain-loss frequency: high-frequency gain (B, D) vs. Low-frequency gains (A, C)) were listed as below table. The result indicated there is non-significant effect in the testing.

| Effect | F | Hypothesis | df | Sig. |
| --- | --- | --- | --- | --- |
| Expected value | 1.77 | 1 | 23 | .20 |
| Gain-loss frequency | 1.56 | 1 | 23 | .22 |
| Expected value * Gain-loss frequency | 0.95 | 1 | 23 | .34 |
