# Supplementary material for: Brain maps of Iowa gambling task
Source: BMC Neurosci. 2008 Jul 26;9:72. doi: 10.1186/1471-2202-9-72 (PMC2518922; doi:10.1186/1471-2202-9-72)
Supplement: Additional file 2 — The learning curve of five blocks in each 20 trials showed that subjects gradually chose the deck B and avoid deck A, but there have no obviously ascending pattern for decks C and D in the IGT. The present result may be inconsistent with the original finding of IGT, but might be congruent with most IGT related studies which did not show the learning curve of each deck (most of them use the combination of good decks or bad decks). The repeated measurement ANOVA for three variables (expected value: bad (A, B) vs. good (C, D), gain-loss frequency: high-frequency gain (B, D) vs. Low-frequency gains (A, C), and block (1–5)) was provided here. The result indicated there is a significant effect on block testing. [file 1471-2202-9-72-S2.doc]

**Additional file 2**

**
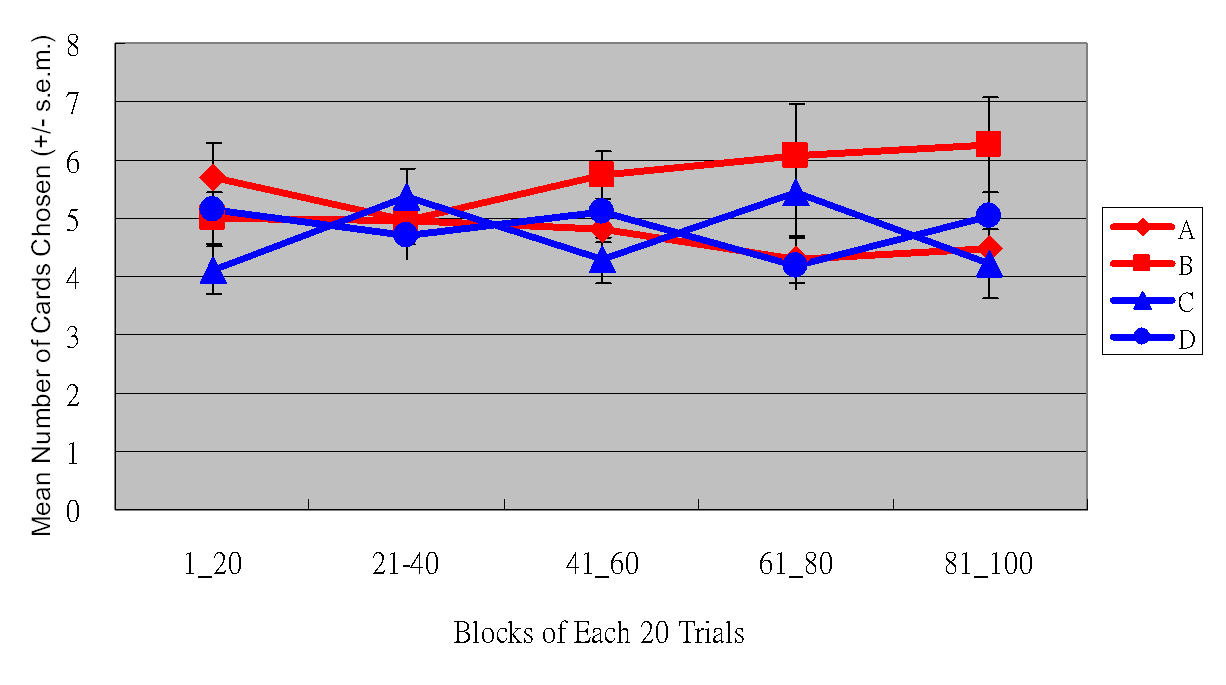
**

The learning curve of five blocks in each 20 trials showed that subjects gradually chose the deck B and avoid deck A, but there have no obviously ascending pattern for decks C and D in the IGT. The present result may be inconsistent with the original finding of IGT, but might be congruent with most IGT related studies which did not show the learning curve of each deck (most of them use the combination of good decks or bad decks). The repeated measurement ANOVA for three variables (expected value: bad (A, B) vs. good (C, D), gain-loss frequency: high-frequency gain (B, D) vs. Low-frequency gains (A, C), and block (1-5)) was provided here. The result indicated there is a significant effect on block testing.

| Effect | F | Hypothesis | df | Sig. |
| --- | --- | --- | --- | --- |
| Expected value | 1.73 | 1 | 23 | .20 |
| Gain-loss frequency | 1.56 | 1 | 23 | .22 |
| Block | 365.58 | 3 | 21 | **.00** |
| Expected value * Gain-loss frequency | .95 | 1 | 23 | .34 |
| Expected value * Block | .55 | 4 | 20 | .70 |
| Gain-loss frequency * Block | 2.261 | 4 | 20 | .10 |
| Expected value * Gain-loss frequency * Block | 2.504 | 4 | 20 | .08 |

Furthermore, the Pair-t test is used to compare the deck B with the other three decks in each block. The statistical table demonstrated that there has a significant effect between deck B and C on block 3.

| **Pair-t test** | **t** | **df** | **Sig. (2-tailed)** |
| --- | --- | --- | --- |
| A1 - B1 | .75 | 23 | .46 |
| A2 - B2 | 0 | 23 | 1 |
| A3 - B3 | -1.62 | 23 | .13 |
| A4 - B4 | -1.60 | 23 | .12 |
| A5 - B5 | -1.55 | 23 | .14 |
| B1 - C1 | 1.29 | 23 | .21 |
| B2 - C2 | -.78 | 23 | .45 |
| B3 - C3 | 2.19 | 23 | **.04** |
| B4 - C4 | .41 | 23 | .69 |
| B5 - C5 | 1.61 | 23 | .12 |
| B1 - D1 | -.33 | 23 | .74 |
| B2 - D2 | .45 | 23 | .66 |
| B3 - D3 | .93 | 23 | .36 |
| B4 - D4 | 1.78 | 23 | .09 |
| B5 - D5 | 1.10 | 23 | .28 |
