# Supplementary material for: Brain maps of Iowa gambling task
Source: BMC Neurosci. 2008 Jul 26;9:72. doi: 10.1186/1471-2202-9-72 (PMC2518922; doi:10.1186/1471-2202-9-72)
Supplement: Additional file 3 — Mean response of brain area with value. The mean activation was further processed from Figure 5, which is the average BOLD signal from the PSTH. The red brackets marked the possible brain regions activated following the monetary change. The lentiform(R) may be in response to the change of positive value. In contrast, the superior temporal gyrus may be sensitive to the change of negative value. [file 1471-2202-9-72-S3.doc]

**Additional file 3**

**
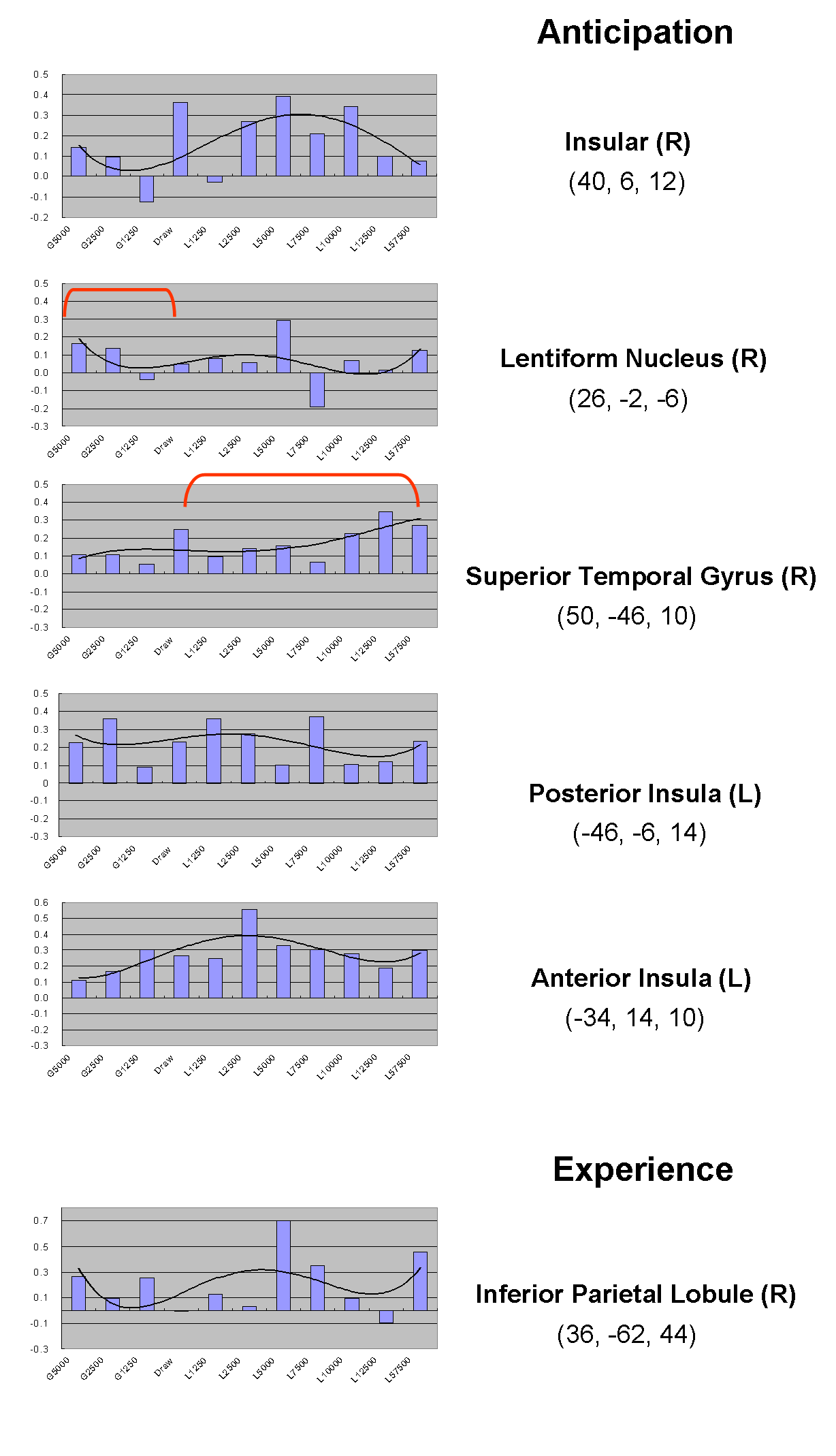
**

**Mean response of brain area with value.** The mean activation was further processed from Figure 5, which is the average BOLD signal from the PSTH. The red brackets marked the possible brain regions activated following the monetary change. The lentiform(R) may be in response to the change of positive value. In contrast, the superior temporal gyrus may be sensitive to the change of negative value.
